# Supplementary material for: UiO-66-NH2 and Zeolite-Templated Carbon Composites for the Degradation and Adsorption of Nerve Agents
Source: Molecules. 2021 Jun 23;26(13):3837. doi: 10.3390/molecules26133837 (PMC8270328; doi:10.3390/molecules26133837)
Supplement: Supplementary file 1 [file molecules-26-03837-s001.zip › molecules-1264277-supplementary.pdf]

Supplementary Material

UiO-66-NH_2 and Zeolite-Templated Carbon Composites for the Degradation and Adsorption of Nerve Agents

Jaeheon Lee, Dongwon Ka, Heesoo Jung, Kyeongmin Cho, Youngho Jin * and Minkun Kim *

Agency for Defense Development, P.O. Box 35, Yuseong-gu, Daejeon 34186, Korea; jhun0740@gmail.com (J.L.); rkehd47@gmail.com (D.K.); hsjung@add.re.kr (H.J.); kmcho@add.re.kr (K.C.)

* Correspondence: cadetnet@add.re.kr (Y.J.); mkkim@add.re.kr (M.K.); Tel.: +82-42-821-2203 (Y.J.); Tel.: +82-42-821-2427 (M.K.)

Table S1. EDS elemental analysis of pristine materials and UiO-66-NH_2/ZTC composites

Element	beta-ZTC	$U_{0.2}Z_{0.8}$	$U_{0.8}Z_{0.2}$	UiO-66-NH_2
**Carbon**	94.4	81.51	33.99	22.07
**Nitrogen**	-	0.01	1.35	1.85
**Oxygen**	5.6	12.17	13.52	13.69
**Zirconium**	-	6.31	51.15	62.39

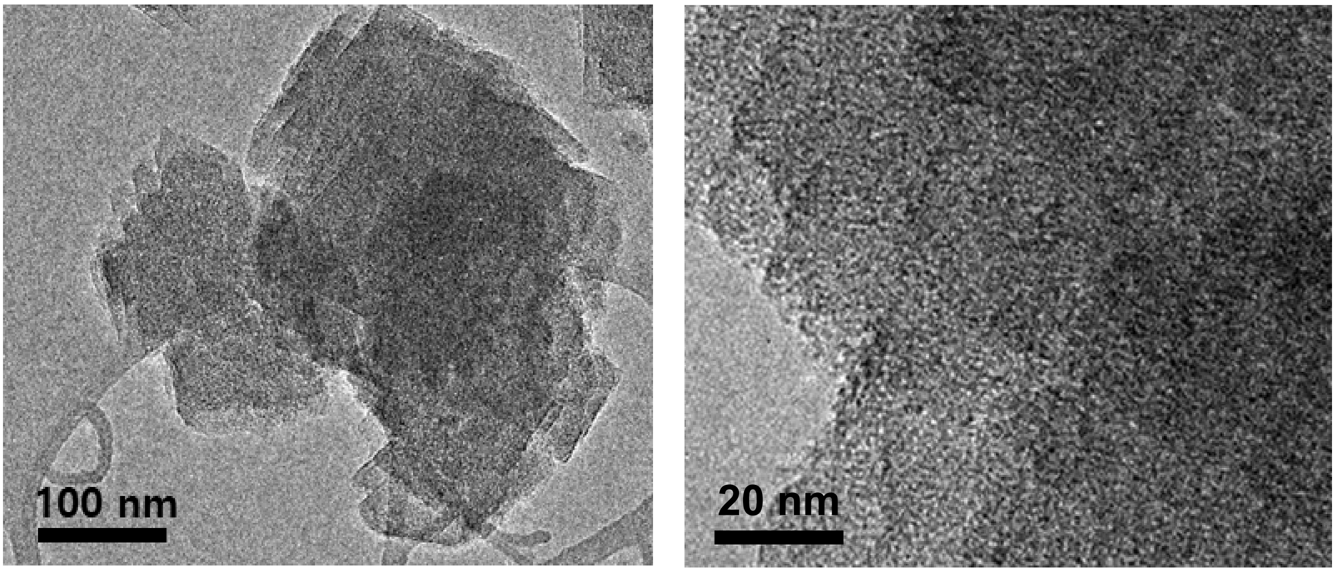

(**a**) (**b**)

Figure S1. TEM images of beta-ZTC in (**a**) low magnification, and (**b)** high magnification.

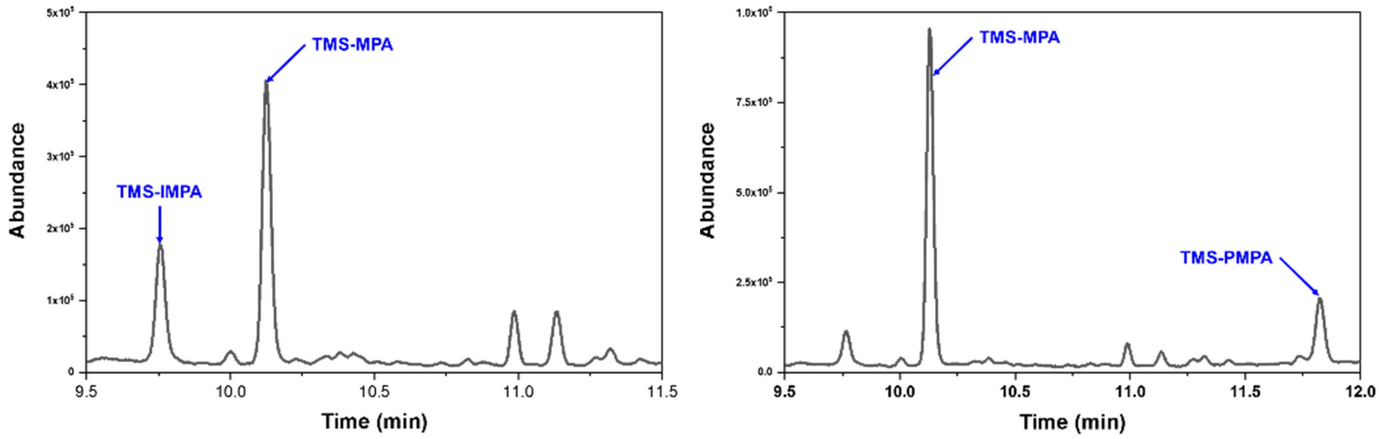

(**a**) (**b**)

Figure S2. GC spectra of the reaction products from the degradation of nerve agents (**a**) GB and (**b**) GD by $U_{0.8}Z_{0.2}$

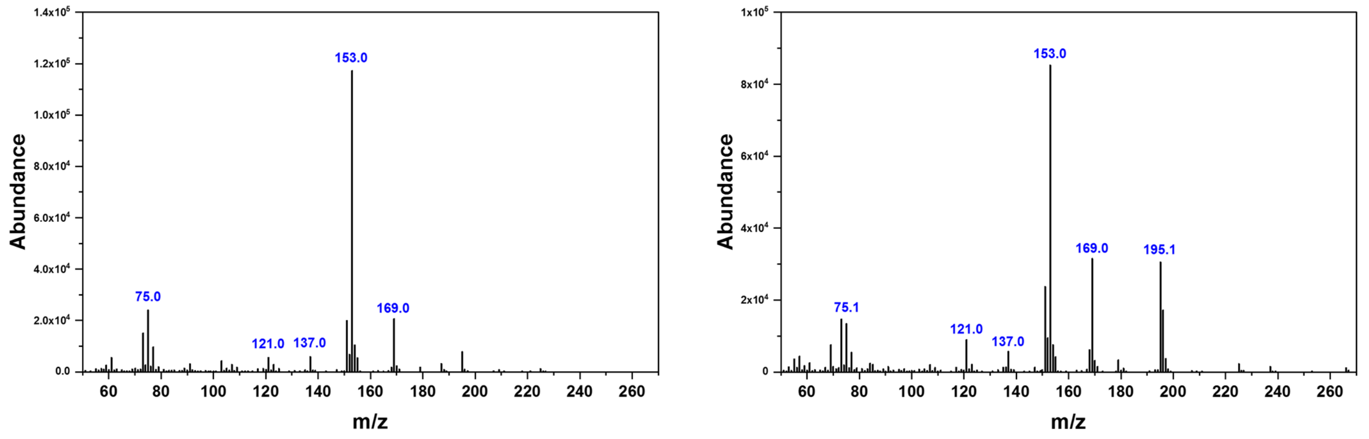

(**a**) (**b**)

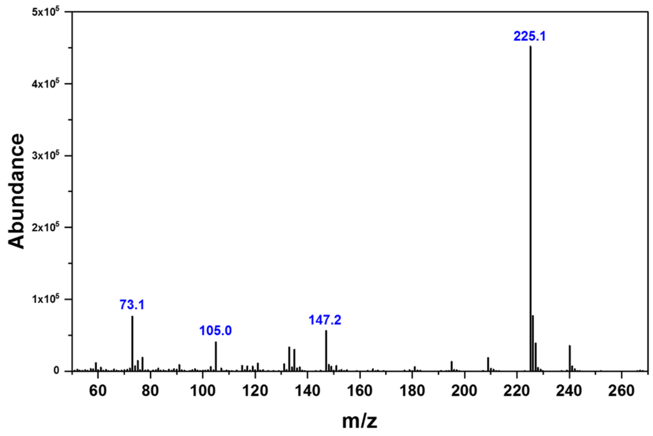

(**c**)

Figure S3. MS spectra of reaction products (a) TMS-IMPA, (b) TMS-PMPA, and (c) TMS-MPA.
